# High Dynamics and Precision Optical Measurement Using a Position Sensitive Detector (PSD) in Reflection-Mode: Application to 2D Object Tracking over a Smart Surface

**DOI:** 10.3390/s121216771

**Published:** 2012-12-06

**Authors:** Ioan Alexandru Ivan, Mihai Ardeleanu, Guillaume J. Laurent

**Affiliations:** 1 FIE&FIMMR, University Valahia of Targoviste, B-dul Unirii Nr.18-20, 130082 Targoviste, Romania; E-Mail: ivan@valahia.ro; 2 FEMTO-ST Institute, UMR CNRS-6174, UFC, ENSMM, UTBM, Dpt. AS2M, 24 Rue Alain Savary, 25000 Besancon, France; E-Mail: guillaume.laurent@femto-st.fr

**Keywords:** microrobotics, optics, optomechatronics, sensing, PSD, position sensitive detector, smart-surface, tracking algorithm

## Abstract

When related to a single and good contrast object or a laser spot, position sensing, or sensitive, detectors (PSDs) have a series of advantages over the classical camera sensors, including a good positioning accuracy for a fast response time and very simple signal conditioning circuits. To test the performance of this kind of sensor for microrobotics, we have made a comparative analysis between a precise but slow video camera and a custom-made fast PSD system applied to the tracking of a diffuse-reflectivity object transported by a pneumatic microconveyor called Smart-Surface. Until now, the fast system dynamics prevented the full control of the smart surface by visual servoing, unless using a very expensive high frame rate camera. We have built and tested a custom and low cost PSD-based embedded circuit, optically connected with a camera to a single objective by means of a beam splitter. A stroboscopic light source enhanced the resolution. The obtained results showed a good linearity and a fast (over 500 frames per second) response time which will enable future closed-loop control by using PSD.

## 1. Introduction

The majority of large and micro scale robotic applications involve high dynamics trajectory control. Rapid sensors for objects positioning are thus required, also combining high accuracy and repeatability. These features are required to attain a precise loop control. The continuous improvement of robotic and micro-robotic applications has been related to the design of optical sensors featuring ever increasing performance.

In the last twenty years the video camera and its related software for image processing became a classical sensorial system for robotics. The visual feedback for robot control is commonly termed as Visual Servoing (VS). The early applications of VS were simple movements and pick-and-place tasks, whereas today VS refers to advanced real-time object manipulation, substantially increasing the flexibility of automation processes [1]. VS represents the result of confluence between important technical areas such as image processing, kinematics and dynamics, theory of control, and real-time computational operations [2].

In VS, the processing delay time needed to extract visual information is the major problem especially for fast dynamic systems, a common case in micro-robotics. Moreover, if several video cameras are required, the computation time required to solve the specific algorithms, increases accordingly. This problem imposes the dynamic limits and the high processing unit costs of most VS applications. According to [1], the VS systems are divided into:

- *position-based*, these systems retrieve the three dimensional information about the scene where a known camera model is used to estimate the position and the orientation of the target with respect to the camera coordinate system;
- *image-based*, 2D image measurements are used directly to estimate the desired movement of the robot;
- “2½*D*” represents a combination of the previous two categories.

In [3] a typical vision-guided robot application, using a video camera integrated into a robot control system, and a simulation platform developed under the Matlab™ environment is presented. Two configurations were described. In the first case the video camera for image capture was mounted on the robot end-effector, in a so-called “eye-in-hand” configuration. In the second case, the camera is fixed at a certain place, called “eye-to-hand” configuration. The system was implemented as a simulator before programming real time vision-guided robot applications, being typical for the deployment of specific algorithms.

A micromanipulation robotic system which operates with micro size objects, presented in [4], integrates a VS-based motion control. The robotic system is composed of two arms equipped with grippers. The VS is of image-based type, and is composed of two orthogonal video cameras, one of them with vertical axis orientation and the second one being horizontal. The system can identify, recognize, track and control moving targets such as micro size objects manipulated on the stage.

The VS systems require complex and top performance hardware and software elements, whose costs are accordingly high. For reasons of simplicity and cost-effectiveness, we have studied and proposed to implement a single object motion tracking system for robotic and micro-robotic applications, industrial or scientific, using a fast and very low cost sensor. This sensor is called a position sensitive (or sensing) detector (PSD) which consists of a special monolithic PIN photodiode with several electrodes in order to achieve detection in 1D or 2D. Compared to discrete element detectors, PSDs have many advantages including high position resolution, fast response time and very simple signal conditioning circuits [5].

Regarding the existing PSD applications, they are quite varied, for instance aeronautics, civil engineering, surgery, robotics, *etc.*, all using light sources (e.g., laser, led) projected by lenses or mirrors over the 1D or 2D surface of the PSD. For instance, an example of an application is related to NASA spacecraft missions (e.g., spacecraft docking) [6]. An important vision-based navigation sensor system (VISNAV) was implemented with a PSD photo-detector in the focal plane of an optical system pointing to a set of light sources or beacons, the line of sight vector between spacecrafts being determined under high-precision relative position conditions.

The high rate of PSD sensors inspired [7] to implement an optical complex system for the x-y detection of elementary particles within an original nuclear microscope (Ion Electron Emission Microscope, IEEM) developed by the SIRAD group at the Legnaro National Laboratory, Padova, Italy. The conclusions of [7] demonstrated the practical limits of PSD *versus* CCD sensors, as both useful and spurious signals were detected by the sensitive area of PSD and the calculation of the tracked particles *versus* the spurious ones was an impossible task.

So far, the robotic applications have usually been designed for running repetitive and tedious tasks in structured environments. However, there exist some complex industrial operations (e.g., grinding, deburring, polishing, manufacturing inspection) that involve the execution of robotic tasks in unstructured frameworks where it is essential to execute unknown trajectories [8]. The specific sensorial systems in these cases are typically cameras or tactile sensors. The PSD is an alternate sensor which offers the possibility to obtain the contours of unknown objects [8]. The authors implemented a control loop in a SCARA experimental robot using a PSD sensor for following a particular shape. A recent paper [9] reported a kinematic Kalman filter sensor fusion of a PSD with an inertial measurement unit. The technique was applied to get a very accurate velocity and position sensing of an industrial robotic arm end-effector.

Another application that integrated PSD was in civil structures engineering for optical recording of the relative-story displacement during earthquakes followed by the remnant deformations measurement of the building structure [10]. The sensor unit was composed of three PSDs and lenses capable of measuring the relative-story displacement successfully.

A major accuracy improvement in microsurgery refers to performing the active tremor compensation within existing robotic hands systems. The authors of [11] developed and tested such a 3D system under experimental conditions. The choice of sensorial elements again balanced the CCD and PSD sensors. After some detailed comparative analysis, the authors of [11] considered the PSD as a suitable sensorial element, offering high accuracy and high frequency response with low cost and complexity.

We conclude that the potential exists for PSDs as cost-effective sensors generating continuous and lag-free data which could be fed into PID controllers with a minimal processing in many fields of activity. The simple conditioning circuits and algorithms can be embedded into microcontroller-based systems, a remarkable technical feature.

Obviously, PSD is not intended to replace all CCD cameras in VS or other technical fields, but may find interesting alternate applications such as the one described in this paper. To test the performance of this kind of sensor for microrobotics, we’ve made a comparative analysis between a precise but slow video camera and a custom-made fast PSD system applied to the tracking of a diffuse-reflectivity object transported by a microconveyor called Smart-Surface. The Smart-Surface micro-robotic application which has been designed by the team of Yahiaoui *et al.*[12,13] at the MN2S department of the FEMTO-ST Institute, France, is a micro-manipulation unit for transporting various small planar objects using air levitation. The principle of objects conveyance supposes cardinally oriented inclined air jets. By combining these orthogonal motion vectors, different planar trajectories may be obtained. Specific MEMS technology was used to fabricate the array of nozzles used for motion vector generation.

The sensorial system used in [13] was a video camera capable of up to 60 frames per second (fps). Provided the fast Smart-Surface dynamics like travel times from border to border was under 100 ms as well as the Firewire 1394 camera bus delay of 15 ms, it was considered as appropriate to upgrade the existing system with a fast PSD by means of an optical beam splitter. The designed PSD system and the first calibration results are presented in the paper. The tracking of a trajectory is depicted, showing positive results.

The paper is structured into five sections. After this first introductory part, Section 2 describes the PSD device and the specific signals conditioning. Section 3 presents the PSD calibration and tracking algorithm based on the four specific PSD signals in referential conditions, and the tracking mathematical calculus. Section 4, called Experiments with a Smart-Surface, describes the Smart-Surface structure, the experimental setup and the data processing results along with a comparative discussion. The conclusions part finalizes the paper by pointing out some future work directions like the closed-loop control.

## 2. PSD Device and Signal Conditioning

The PSD is a silicon optical device which has the capacity to convert an incident light spot position into a series of analogue signals. The property to generate continuous data represents the basic feature and advantage of a PSD. According to surface structure, the PSD detectors may be divided into two classes: segmented or isotropic. The segmented detectors are made of independent elements and require broader light spots. For instance four-quadrant photodiodes are commonly used in atomic force microscopy position control feedback. The isotropic PSDs consist of a single active element and are based on the lateral photovoltaic effect. Depending on the patterned electrodes, the isotropic PSDs are divided into two sub-classes: linear and two-dimensional. The latter category is manufactured either with four pins (two anodes and two cathodes) or with five pins (one cathode and four anodes).The isotropic PSDs show a very good continuity (no dead spaces) and are robust with respect to the spot size and the image focusing. All types of PSDs show very fast response times, in the microsecond range. Other important features are their good resolution and linearity over a wide range of light intensities.

**Figure 1.**
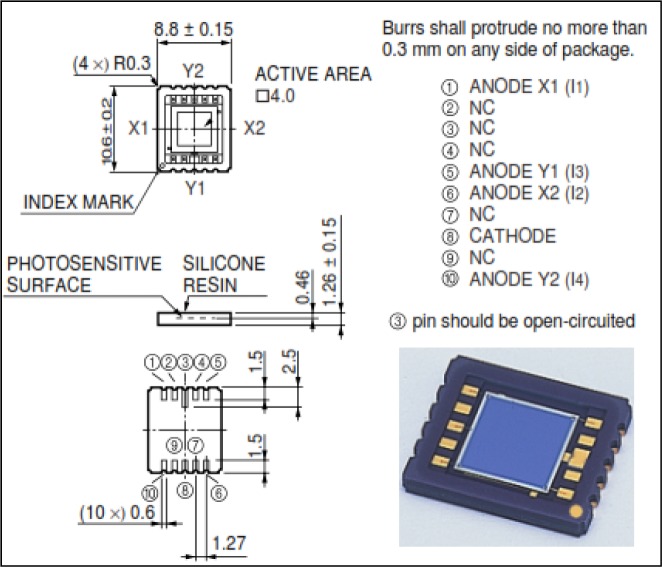
PSD unit Hamamatsu S5990-01, datasheet from [5].

In Figure 1 the constructive details regarding distinct sensing electrodes are presented. The output analogue signals depend on the distance between the corners and the light spot position (more exactly the position refers to the centre of the incident spot light). The PSD primary signal processing involves signal amplification, analog-to-digital conversion and calculation using standard formulae like the ones described in Equations (1–3).

We used a commercial Hamamatsu PSD unit model no. S5990-01 featuring a two-dimensional square 4 × 4 mm^2^ sensing area, as depicted in Figure 1. The manufacturer formula for the *x-y* coordinates of a laser or LED spot focused on the surface is:

(1) $$x=\frac{L}{2}\times \frac{\left(I_{2}+I_{3})-(I_{1}+I_{4}\right)}{I_{1}+I_{2}+I_{3}+I_{4}}\text{scale}\;\text{equations}\;\text{the}\;\text{same}\;(\text{approx}.\;\;12\text{pt})$$

(2) $$y=\frac{L}{2}\times \frac{\left(I_{2}+I_{4})-(I_{1}+I_{3}\right)}{I_{1}+I_{2}+I_{3}+I_{4}}$$

where L is the side of the square (4.5 mm) and *I*_1_ to *I*_4_ are the values of the four anode currents (see Figure 1).

**Figure 2.**
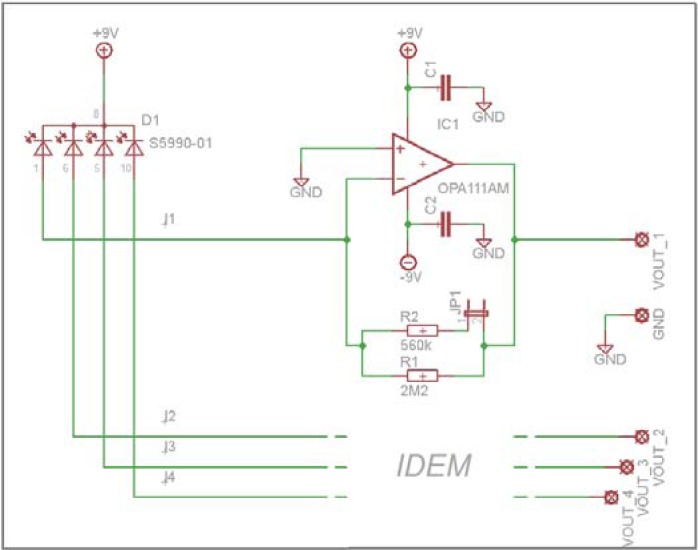
The PSD electronic circuit schematics.

An electronic circuit with four transimpedance amplifiers has been designed (Figure 2) to convert and amplify the *I*_1_ to *I*_4_ signals. It is based on a single-stage schematic featured with low noise and low bias currents op-amps (type OPA111). The output signals are:

(3) $$V_{\mathrm{out}\_1...4}=-R\cdot I_{1...4}$$

where *R* are the feedback resistances which, in our case took two values: 2.2 MΩ (jumper off) for a high gain or 446 KΩ (jumper on) for a lower gain. This two-stage gain was implemented due to the initial uncertainty related to the received amount on light. The reported results in the following sections are all recorded with the high gain.

**Figure 3.**
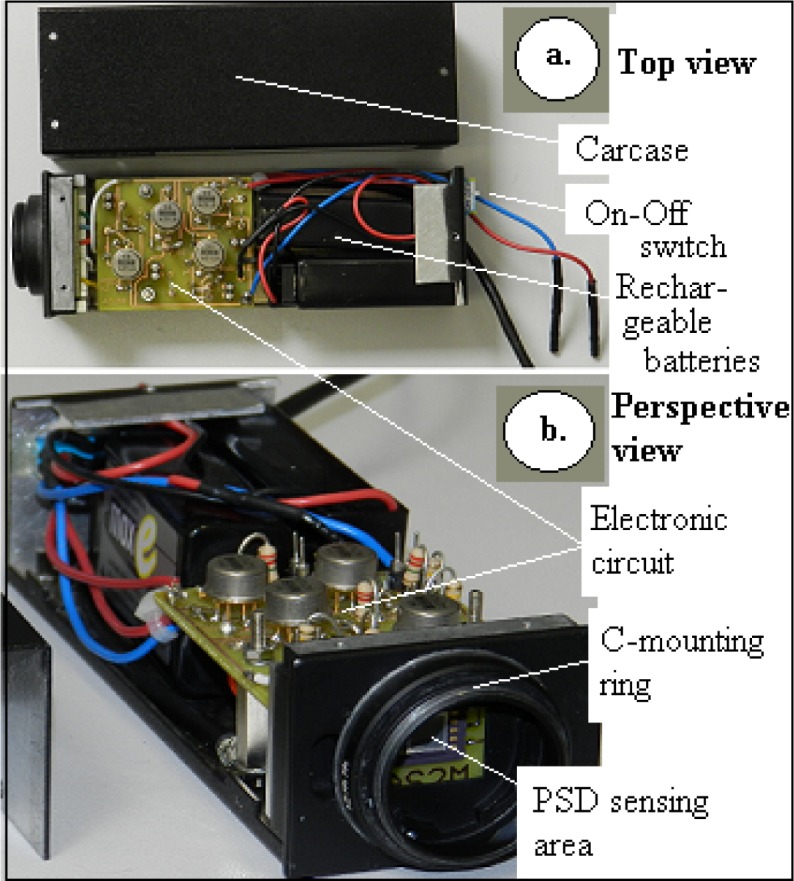
The custom-made PSD circuit featuring a C-mount standard ring and an embedded electrical supply.

The complete and custom-manufactured PSD circuit can be seen in Figure 3. The system includes the PSD sensor (front view), the amplifier circuit, four jumpers for the High/Low gain selection, the batteries and an On/Off switch. The C mounting ring insures the device adaptation to the optical axis. The device electrical source was composed of two embedded 9V DC rechargeable batteries providing over 10 hours autonomy. They have been embedded in order to get the best achievable EMF noise protection—a critical aspect for such low amplitude signals.

## 3. PSD Calibration Algorithm

**Figure 4.**
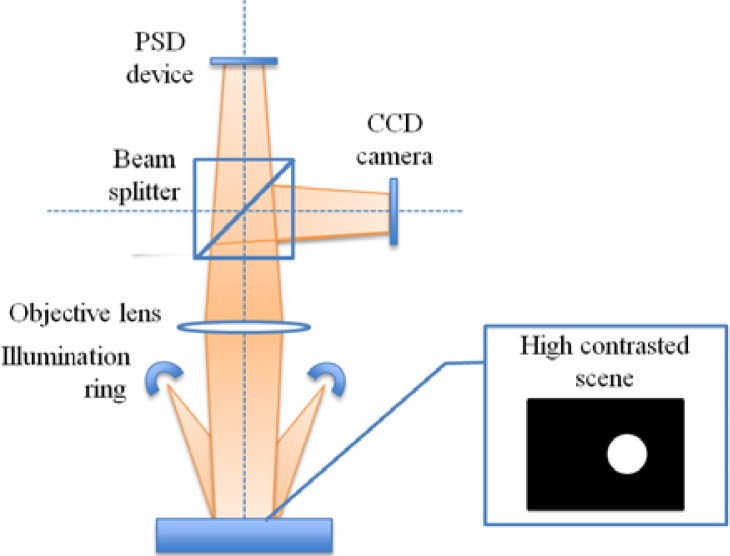
The optical elements of the PSD system operating in diffuse reflectivity mode. The beam splitter and the CCD camera are for calibration purposes.

Unlike the typical applications where a light spot is focused on the PSD surface, the actual system was designed to work in the diffuse reflectivity mode, where the object is projected over the PSD surface like in the schematic shown in Figure 4. Under epi-illumination, the white matt object will appear very bright against the highly reflective silicon Smart-Surface which shall appear dark. Despite that contrast, the actual amount of incident light returned to the PSD is very small, with the resulting currents ranging around 0.1 μA, depending on the object reflectivity and on the lens *f*-ratio. Therefore, we chose a stroboscopic lighting based on a monostable circuit, which, as will be shown, successfully improves the signal-to noise ratio. The monostable circuit has been designed with the classic NE555, an integrated circuit suitable for a panel of timer applications and basically operating by charging and discharging an external RC network.

**Figure 5.**
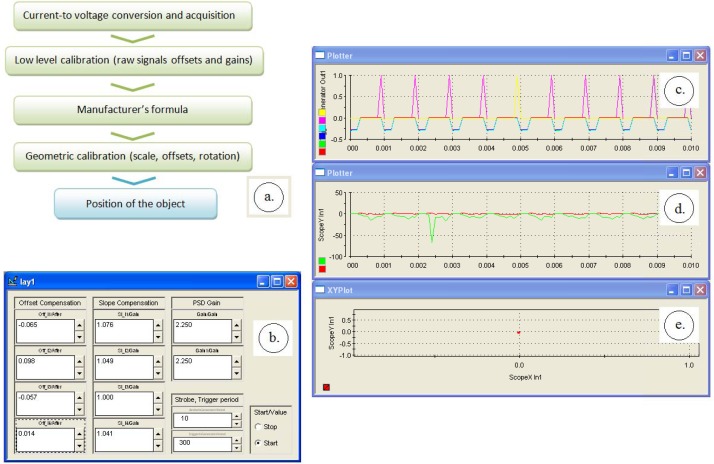
The calibration precedure: (**a**) shows the conversion sequence block diagram (**b**) shows the initial calibration window of the four raw PSD signals, (**c**) shows the raw plot of the four PSD signals as well as the stroboscopic digital impulse train (**d**) contains the raw *x*&*y* charts and (**e**) is the final *x-y* graph of filtered values (the PSD spot).

There are four steps to get the calibrated *x-y* position (Figure 5(a)): the initial current-to-voltage and analog-to-digital conversion, a low-level calibration, the conversion formula and the secondary calibration. The low level PSD calibration supposes equalizing the *V_OUT__*_1_ to *V_OUT__*_4_ signals. That is to be performed over a flat gray surface with the stroboscopic lighting on and by adjusting the offset and slope parameters. Figure 5(b) shows the dedicated interface which allowed the manually equalizing of the four raw signals.

After the raw signals calibration, the device is ready to acquire the *x-y* data by applying the formulas (1–2). This *x-y* data are however not reliable yet because they require further calibration by means of a reference camera or by positioning the object to a set of precisely known places. We chose the first solution, the reference position being provided by a camera connected to the same optical lens by means of a beamsplitter. The *x-y* signals calibration involve adjusting a gain parameter and some offset and rotation parameters between the two coordinate axis systems of the PSD and of the camera. The formula which providing the final, corrected *x_C_-y_C_* coordinates, is:

(4) $$\left(\begin{array}{c} x_{C}\\ y_{C} \end{array}\right)=\left(\begin{array}{c} x_{0}\\ y_{0} \end{array}\right)+G\left(\begin{array}{ll} \text{cos}\phi & -\text{sin}\phi \\ \text{sin}\phi & \text{cos}\phi \end{array}\right)\left(\begin{array}{c} x\\ y \end{array}\right)$$

where *x* and *y* are the raw coordinates provided by Equations (1–2), *G* is an adjusting slope parameter, *x_0_* and *y_0_* are the translation offset parameters and *φ* is the rotation angle between the two coordinate systems. The identification of these four parameters acting in Equation (4) is performed initially once, by arbitrarily displacing the object in two diagonal points and by recording the *x-y* PSD coordinates as well as the corresponding CCD camera images reference *x_R_-y_R_* coordinates, resulting thus a simple system of four equations with four unknowns (*x_0_*, *y_0_*, *G* and *φ*). The reference *x_R_-y_R_* coordinates are calculated under Matlab^™^ by applying simple centroid functions to the recorded CCD images (further details are provided in Section 4).

**Figure 6.**
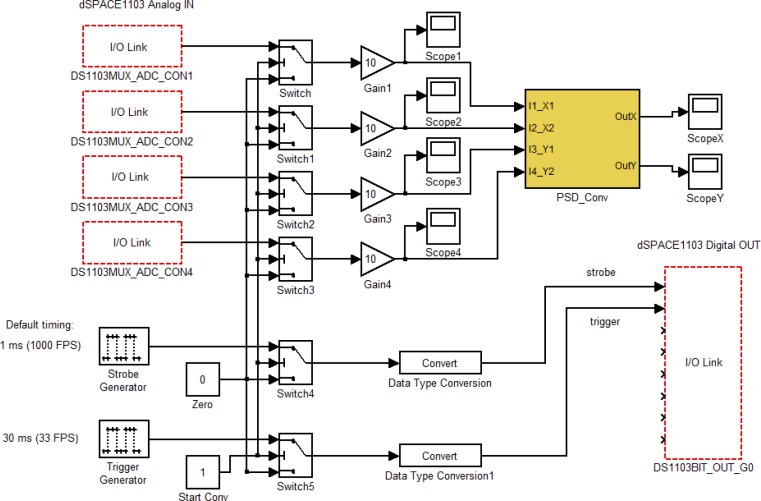
The Simulink model for position sensing, embedded into the real time dSPACE board. The *PSD_Conv* block contains the formulas (1), (2) and (4).

The formulas (1–2) and (4) have been implemented in a Simulink model as shown in Figure 6 and deployed on a dSPACE data acquisition and control board. The PSD instantaneous coordinates *x_C_-y_C_* are thus computed in real time, allowing fast object tracking. As said, the system uses stroboscopic lighting which is generated by the same dSPACE system. Finally, the external camera is also synchronized with the algorithm, by connecting a digital port of the dSPACE machine to the external trigger input of the camera. The results are presented in the next section.

## 4. Experiments with a Smart-Surface

The Smart-Surface is a pneumatic device conceived for small objects manipulation by using the air levitation. The device array is a 9 mm × 9 mm square and is composed by 8 × 8 pneumatic micro-conveyors. A micro-conveyor has four nozzles able to generate inclined air-jets in the four cardinal directions. An object of small weight and small dimensions, can be conveyed to a desired position with intermittent air-flow pulses of a pre-adjustable pressure up to 20 kPa [13]. The used experimental object is a disk of 3.5 millimetres diameter and 12 milligrams weight. These physical features were chosen from dynamic considerations.

**Figure 7.**
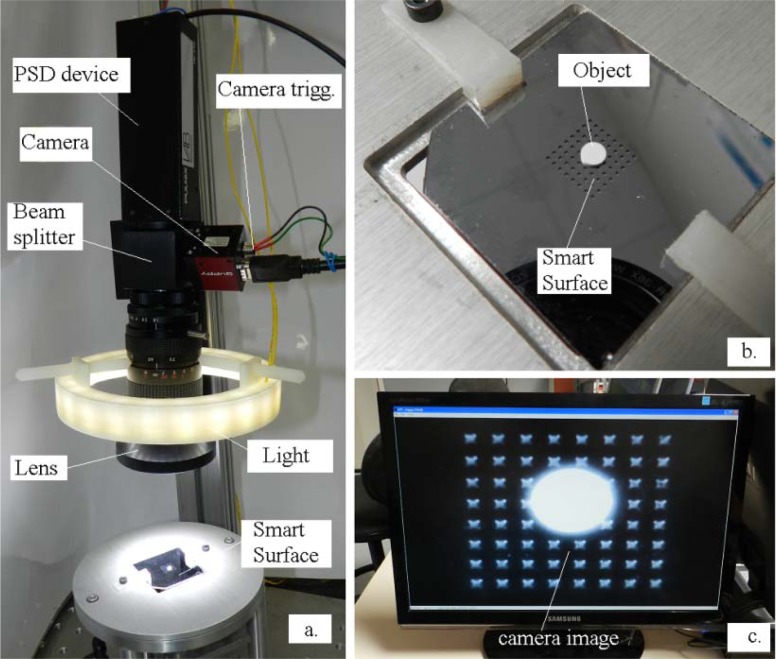
Smart-Surface and sensory system. (**a**) General system view. (**b**) Zoom of the Smart-Surface. (**c**) Camera capture.

Figure 7(a) shows the main elements of the application. The camera model is an IEEE 1394 bus AVT Guppy model equipped with a 1/2” (6.4 × 4.8 mm^2^) monochrome sensor. The focusable TV lens specifications are: 12.5–75 mm, f/1.8, C-mount. The equivalent system focal length is however altered because of the added beamsplitter cube of 38 mm. The Smart-Surface and its corresponding object are presented in Figure 7(b). The scene is epi-illuminated with a number of 24 high efficiency 5 mm white LEDs positioned in three parallel rows. The camera source image through the optical system is shown in Figure 7(c). This is the acquired image which is processed to get reference coordinates.

**Figure 8.**
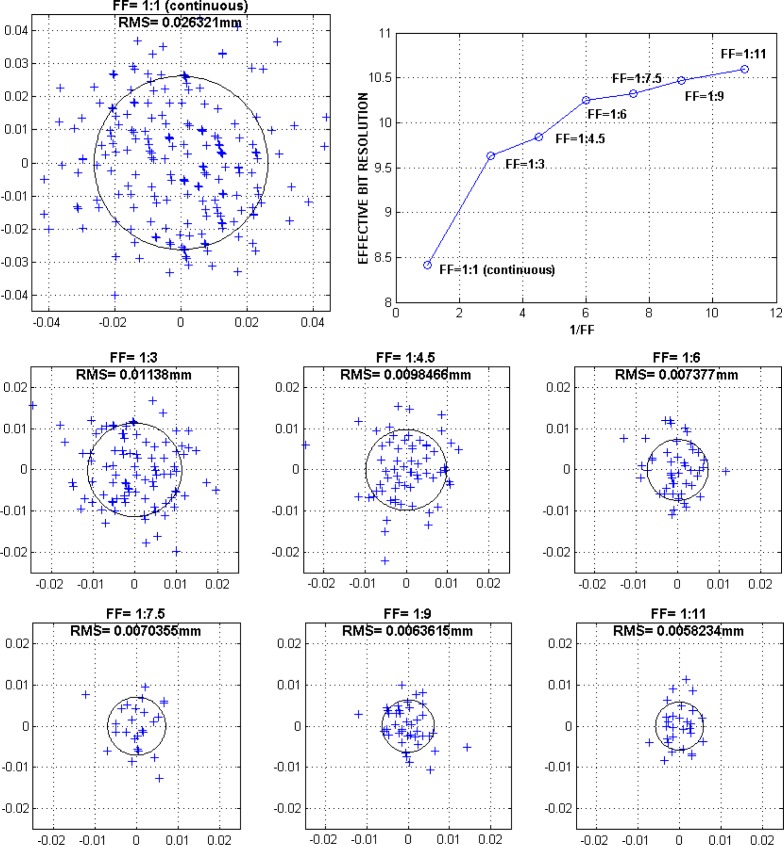
Spot dispersion root mean square (RMS) under different Fill Factor (FF) strobe lighting conditions. The experimental points are marked with crosses. The RMS values are represented numerically and graphically (the corresponding circle radius). All units are in mm. The LED average current has been kept constant to the nominal value of 0.27 A. Strobe light pulse was kept constant (0.33 ms) while the dark period was varied in order to tune the FF. The PSD system resolution expressed in effective bits is starting from 8.5 bits (continuous or DC lighting) and saturating towards 10.6 bits (FF = 1:11).

The Smart-Surface is lighted by strobed pulses; their higher intensity leading to better signal-to-noise ratios. To determine the light pulses optimum fill factor (FF), or duty cycle, which is the ratio of the pulse duration to the total period, it is necessary to evaluate the dispersion of the PSD measurement points with the variation of this parameter. Figure 8 shows the numerical dispersion results in terms of root mean square (RMS) values corresponding to DC (direct current) and to a series of fill factors ranging from 1:3 to 1:11. During experiments the average (light and dark) supply current was kept to the nominal value of 0.27 A. The net improvement in terms of effective bits resolution is straightforward. We may observe that fill factor FF = 1:11 provide the best results with a corresponding RMS value of 5.8 μm. However, we may notice a saturation above FF = 1:11. Better effective resolutions could be probably achieved only by upgrading the lighting ring intensity.

**Figure 9.**
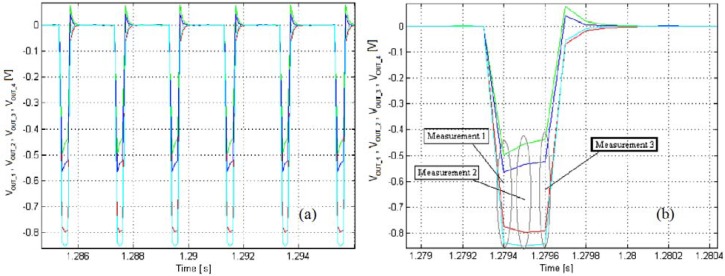
(**a**) The PSD four typical raw waveforms. Strobed pulses start on the falling edges. (**b**) Zoom of the acquired waveforms, during a light impulse.

For the following experiments we selected a lighting strobe frequency of 500 Hz and a fill factor FF = 1:6. The slower camera trigger has been configured for a frame rate of 33.3 fps, which corresponds to an image acquisition every 15 light shots. The dSPACE Simulink has been configured with a sample period of 10^−4^ s, which means a number of three data samples for every 0.33 ms light impulse. Figure 9 shows the typical *V_out_1...4_* input waveforms. As may be observed from the zoomed Figure 9(b), the curves are slightly transient; therefore, only the 3rd measurement point (in bold case in the figure) is selected for the *x-y* conversion, the other points being ignored by the tracking algorithm.

**Figure 10.**
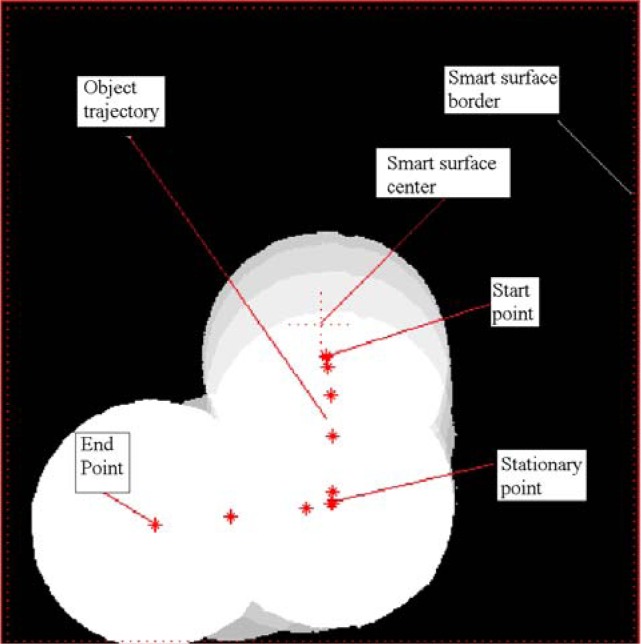
Construction of the reference trajectory using image recording every 30 ms and subsequent off-line processing.

As stated, the camera acquisition period is of 30 ms, precisely triggered each 15th light pulse. The frames are recorded individually and processed off-line for evaluation under Matlab™. The image processing consists of two steps. First, the acquired image is thresholded to get a binary image. The value of the threshold is the median value between the intensity of the background and the intensity of the object. Then, the object is tracked using a binary-large object (BLOB) extraction approach. The position of the center of the object is computed as the centroid of the BLOB to get accurate sub-pixel position. The obtained frames are displayed like in Figure 10.

**Figure 11.**
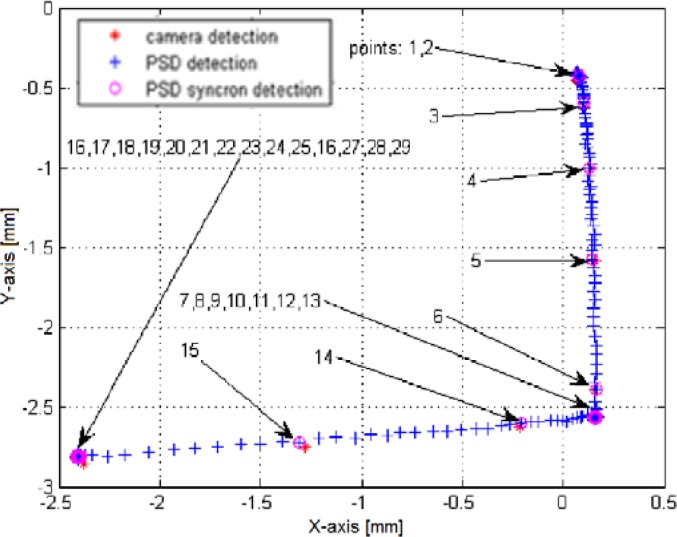
The superposition of PSD and camera trajectories shows a very good correlation.

**Figure 12.**
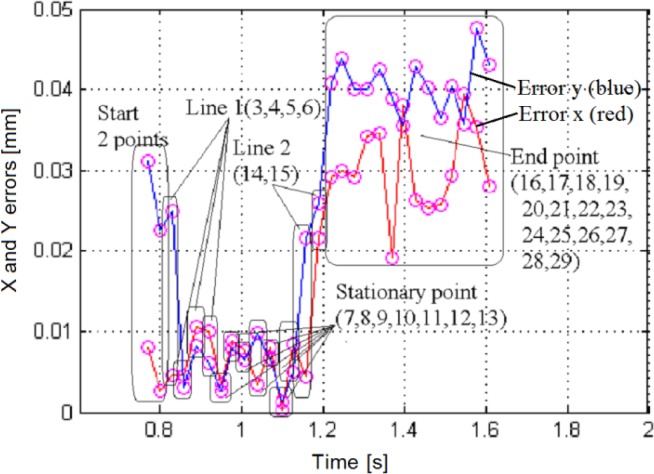
Plot of the absolute x and y errors related to the trajectory of the Figure 11. The maximum error is less than 50 μm.

Figure 11 displays the superposed datasets issued from both the PSD and the camera. The imposed trajectory is of an inverted ”L” shape, brief air bursts being applied in –Y then –X direction. The object acceleration is slower than its deceleration. A linearity deviation of ∼5° can be noticed, which is somehow fair for this type of pneumatic actuation [13]. The superposition of the two trajectories shows a very good agreement. The *x* and *y* errors are inferior to 50 μm, as shown in Figure 12. This corresponds to a relative error of less than 1.5% over the entire measurement range, making the method highly suitable for further closed-loop applications.

## 5. Conclusions and Perspectives

Position sensitive detectors (PSDs) are analogue optical sensors featuring fast response times and simple electronic circuitry. These devices have been used in a various applications ranging from space navigation to surgery, all involving a laser or other direct light spots projected over the PSD surface. The proposed approach consisted of a rather different and indirect mode, by focusing the reflected light issued from a diffuse-reflectivity object on a PSD, whose object coordinates could be thus evaluated.

The designed system is considered as a replacement for the expensive cameras tracking single objects of good contrast and fast dynamics. Some typical applications suitable for the designed system can be found in microrobotics. To test the performance of this kind of sensor for microrobotics, we have made a comparative analysis between a precise but slow video camera and a custom-made fast PSD system applied to the tracking of a diffuse-reflectivity object transported by a pneumatic microconveyor called Smart-Surface. The fast system dynamics prevented until now the full control of the smart surface by visual servoing, unless using a very expensive high frame rate camera.

The system consisted of a PSD plus its battery supply and low noise signal conditioning circuitry, all embedded in a single case provided with a universal C-mount adapter. An optical beamsplitter was mounted between the PSD and the focusable objective provided an additional optical port for a reference camera. Because of the small amount of effectively returned light, a stroboscopic lighting system has been designed in order to enhance the system resolution. The system was controlled and synchronized by a dSPACE real time acquisition and processing unit.

The tests were performed at a framerate of 500 fps for the PSD and confronted with a slower reference camera of 33.3 fps. The calibration procedure was linear, involving the adjustment of gain, slope and rotation angle. There has been found a good agreement between the PSD and the camera trajectories, the maximum relative error being less than 1.5% over the measurement range. Using strobed light, the detection resolution dropped to 6μm RMS.

The results demonstrate the suitability of the PSD system for the closed-loop control. Several perspectives arise. Provided sensing algorithm simplicity, the associated control could be deployed on the same basic microcontroller unit, thus resulting a completely-embedded system. The Hamamatsu S5990-01 response time is of 1 μs, faster frame rates are thus readily achievable. A better concentrated, higher intensity lighting source of 980 nm wavelength would further improve the signal-to-noise ratio. Several other enhancements could be performed such as an automatic gain control (AGC) for the input signals and a semi-automated, assisted *x-y* calibration procedure. By using a quadratic (or a higher order) polynomial calibration or a look-up table interpolation [9], the net accuracy is expected to increase by a factor of 10. Over that value several other limitations arise such as the lens distortion, the finite reference camera resolution (subpixel algorithms required), the electronic noise (better lighting and reflectivity, additional filters), the surface property (avoiding dust particle contamination of PSD and scene) *etc.*
